# Reconstruction of the ZnAl Mixed Oxides Into the Layered Double Hydroxide Catalysts Active in the Aldol Condensation of Furfural: The Role of ZnO Particles

**DOI:** 10.3389/fchem.2021.803764

**Published:** 2022-01-14

**Authors:** Lada Dubnová, Rostislav Daňhel, Vendula Meinhardová, Valeriia Korolova, Lucie Smoláková, Tomasz Kondratowicz, Oleg Kikhtyanin, Libor Čapek

**Affiliations:** ^1^ Department of Physical Chemistry, Faculty of Chemical Technology, University of Pardubice, Pardubice, Czechia; ^2^ Technopark Kralupy VŠCHT Praha, University of Chemistry and Technology Prague, Prague, Czechia

**Keywords:** ZnAl reconstructed LDHs, temperature programmed techniques, *in-situ* DRS, structural properties, basic properties, Na leaching, aldol condensation of furfural

## Abstract

A memory effect is the ability to restore the original, lamellar layered double hydroxide structure. Herein, we have described 1) the changes in the structural and basic properties of ZnAl mixed oxides during their transformation into ZnAl-reconstructed LDHs (RE-LDHs); 2) the extraordinary properties of ZnAl RE-LDHs compared to the original ZnAl LDHs; and 3) the changes of basic properties during the interaction of ZnAl RE-LDHs with atmospheric CO_2_. Aldol condensation was selected as probe reaction to prove the catalytic potential of ZnAl RE-LDHs. We have described a target method for preparing ZnAl RE-LDHs with a large number of basic sites. ZnAl RE-LDHs possess significantly higher furfural conversion in the aldol condensation of furfural than MOs. The structural, textural, and basic properties of the studied materials were described by temperature-programmed analysis, X-ray diffraction, N_2_ adsorption, temperature-programmed desorption of CO_2_, and *in-situ* diffuse reflectance spectroscopy.

## Introduction

Layered double hydroxides (LDHs) are the members of a group of layered materials comprising divalent (Mg, Zn, Ni, Cu, and Co) or trivalent (Al, Ga, and Fe) metal cations. A net positive charge in an LDH crystal lattice is compensated by extra-framework charge-balancing anions, generally carbonates (Bukhtiyarova, 2019). A distinctive feature of LDHs is their supposed memory effect. After a thermal treatment for the transformation of LDHs into mixed oxides, it is possible to recover the original layered structure by rehydrating the mixed oxides in decarbonylated water (Debecker et al., 2009; Sikander et al., 2017).

We focused on ZnAl-reconstructed LDHs (RE-LDHs). In general, ZnM LDHs (M = Al^3+^, Fe^3+^, Ga^3+^, and Ti^3+^) (Palmer et al., 2011; Wang and Zhang, 2012; Kikhtyanin et al., 2018; Bukhtiyarova, 2019; Santamaría et al., 2020; Szabados et al., 2020) can be prepared by several methods from various precursors of given metals, such as nitrates (Bellezza et al., 2014; Zeng et al., 2017; Tang et al., 2019), chlorides (Ambrogi et al., 2012; Zhang and Li, 2014) or sulfates (Mishra et al., 2017).

The most widely used method for the preparation of ZnM LDHs is the coprecipitation of the solutions of salts in different Zn/M ion ratios in a basic medium, where the pH of the medium is maintained and the given materials are precipitated during synthesis. The basic buffer can be Na_2_CO_3_ (Jiang et al., 2010; Kumar and Pant, 2020), NaOH (Nishimura et al., 2013; Tang et al., 2019), any combination thereof (Smoláková et al., 2011; Zeng et al., 2017; Liu and Yang, 2018; Teodorescu et al., 2020), or other solutions that are not widely used, such as a mixture of either Na_2_CO_3_ and (NH_4_)_2_CO_3_ (Korošec et al., 2020) or urea, Na_2_CO_3_, and NaOH (Gil et al., 2020) or aqueous ammonia solution (Bellezza et al., 2014). The quality of the resultant product depends on the pH, which must be maintained during synthesis. If the pH is low during the preparation, not all the ions precipitate out of the solution. On the contrary, at high pH values, the metal ions could dissolve. The chosen pH depends on the use of individual cation types (Sikander et al., 2017). For the preparation of ZnM LDHs, the pH is typically maintained between 9 and 10 (Gil et al., 2020; Santamaría et al., 2020; Dat et al., 2021). The resultant material is washed to remove any residual substances such as Na^+^. Other important parameters of the synthesis are the reaction temperature, concentration, dosing of reactants, stirring of the resultant mixture, and maturation time of the formed precipitate (Jiang et al., 2010).

A method involving urea hydrolysation is used to prepare ZnM LDHs (Montanari et al., 2010; Teruel et al., 2010; Suárez-Quezada et al., 2019; Sakr et al., 2021). Metal salts are dissolved in urea, which hydrolyses extremely slowly. Thus, the resulting material precipitates slower than that in the coprecipitation method. The main advantage of this method is the considerably easy washing of the prepared LDH during synthesis, since there is no need to remove alkali metal ions, as compared to the use of Na_2_CO_3_ or NaOH (Suárez-Quezada et al., 2019). This method can be used to prepare LDHs with a narrow particle distribution and good crystallite size (Liu et al., 2014).

A mechanical–chemical method (Hernández et al., 2017), where the oxides of the respective metals are mixed, and a sol–gel method (Valeikiene et al., 2019), where the precursor salts of the metals or their organic compounds are hydrolysed in water or other organic solvents, were also used.

Heat treatment of the as-prepared ZnAl-LDH yields the corresponding ZnAl mixed oxide. The thermal treatment of LDH results in the removal of physically adsorbed substances and structural water at low temperatures (below 200°C), while the structure of LDH is unchanged. At high temperatures, the LDH structure is dehydroxylated, and ZnO nuclei doped with Al^3+^ are formed as an amorphous phase followed by the formation of homogeneously dispersed ZnO nanoparticles (Zhao et al., 2010; Smolakova et al., 2018). The transformation of LDH to a mixed oxide depends on many parameters, such as the anion type, material pre-treatment, and particularly the Zn/Al ratio (Kovanda et al., 2010). One advantage of ZnAl mixed oxides is their large specific surface area and associated accessibility of active sites (Xu et al., 2013; Hernández et al., 2017). ZnM mixed oxides are used as catalysts in reactions, such as the aldol condensation of furfural (Hernández et al., 2017; Smoláková et al., 2017a; Smolakova et al., 2018), the oxidative dehydrogenation of ethane or propane (Smoláková et al., 2011), alkylation (Grabowska et al., 2001), the steam reforming of methanol (Hammoud et al., 2015) or biogas dry reforming (Calgaro and Perez-Lopez, 2019), and transesterification (Smoláková et al., 2017b; Jadhav et al., 2020). In addition, they are used as photocatalysts (Sescu et al., 2020) and semiconductors for solar cells (Teruel et al., 2010) and CO_2_ adsorbents (Rossi et al., 2016).

RE-LDHs are characterised by a greater activity of the given materials in base-catalysed reactions than in the original LDHs (Kikhtyanin et al., 2018). The reason for this increased activity could be a change in the basic properties of RE-LDHs associated with the formation of additional Brønsted basic OH^−^ sites in the interlayer space instead of the original CO_3_^2−^ anions that are contained in the original LDHs and are released from materials during calcination to mixed oxides (Smoláková et al., 2017a)., Lewis basic sites (medium Zn–O and Al–O and strong O^2−^ sites) of the mixed oxide change to Brønsted basic sites (typically OH^−^), which are incorporated into the layers of RE-LDHs (Álvarez et al., 2013; Hernández et al., 2017).

ZnAl RE-LDHs can be prepared by mixing a composite oxide with decarboxylated water (Hernández et al., 2017). The resulting material is subsequently dried in air, under vacuum, or an inert atmosphere, which prevents undesired CO_2_ chemisorption. In addition, RE-LDHs can be prepared by rehydrating the mixed oxide in an inert gas stream of decarbonated water vapour (rehydration in the gas phase), as reported for MgAl RE-LDHs (Abelló et al., 2005). All reconstructed materials are highly susceptible to the adsorption of atmospheric CO_2_, which results in a decrease in the efficiency of the catalytic activity (Abelló et al., 2005; Xu et al., 2013). ZnAl RE-LDHs can also be prepared the hydrothermal reconstructing route (Tajuddin et al., 2019), and the anion exchange rate, e.g. in the presence of NaCl solution (Kang and Park, 2022).

To prevent CO_2_ adsorption on RE-LDHs, the formation of RE-LDHs could be directly performed in a reaction mixture containing water. MgZn/Al RE-LDHs have been used in epoxidation (Angelescu et al., 2008); however, ZnAl RE-LDHs have not been widely studied and have not been applied as much as MgAl RE-LDHs are applied. MgAl RE-LDHs are used in aldol condensation (Abelló et al., 2005; Xu et al., 2013; Hernández et al., 2017; Horáček et al., 2021) or isomerisation (Debecker et al., 2009; Kwon et al., 2020) and other reactions such as styrene epoxidation (Chimentao et al., 2007), transesterification (Zeng et al., 2014; Dahdah et al., 2021), steam reforming (Dahdah et al., 2020),methanolysis (Navajas et al., 2018), Knoevenagel and Claisen–Schmidt condensation (Abelló et al., 2005) and Michael addition (Abelló et al., 2005).

Aldol condensation of furfural and acetone is an interesting and an important reaction from many aspects. From practical point of view this reaction provides a possibility to construct complex organic molecules with increased value starting from simple and biomass-derived ones. Aldol condensation is also a suitable reaction that allows probing the basic properties of solids, especially RE-LDHs. Currently, there are no reliable instrumental methods to characterize Broensted basic sites in RE-LDHs. Indeed, the most common TPD-CO_2_ is not operative for these materials, because CO_2_ is not adsorbed on basic sites but chemically react with the exchangeable hydroxyls in the RE-LDHs which are Broensted basic sites and transform them into carbonates.

This study aims to reconstruction ability of ZnAl MOs to ZnAl RE-LDHs under their rehydration and to describe the changes in the structural and basic properties of ZnAl mixed oxides during their transformation into RE-LDHs and the relationship between the properties observed for the ZnAl RE-LDHs, as-prepared ZnAl LDHs, and ZnAl mixed oxides. In principle, ZnAl MOs possess less amount of basic sites than MgAl MOs. It is due to basic properties of MgO as a main constituent component in the MgAl MOs. However, the basic properties of the ZnAl RE-LDHs are much less evident, because they are originated from ZnO, i.e. host oxide with lower basic properties. This study shows the formation of basic sites in ZnAl RE-LDHs. ZnAl RE-LDHs with various properties were prepared by three different syntheses of the original ZnAl LDHs: Jiang et al. (2010), Kovanda et al. (2010), Liu et al. (2014). This follows our previous study describing the transformation of ZnAl-LDH into corresponding ZnAl mixed oxides (Smolakova et al., 2018) and the catalytic behaviour of ZnAl mixed oxides in the aldol condensation of furfural (Dubnová et al., 2021).

## Experimental Process

### Preparation of ZnAl Materials

ZnAl-X-HT (X = Zn/Al molar ratio, determined by X-ray fluorescence (XRF) analysis) LDHs with different Zn/Al molar ratios (1–5) were prepared by coprecipitation using an aqueous Na_2_CO_3_ solution as a buffer, following the method described by Jiang et al. (2010). ZnAl-1.9K-HT (Zn/Al molar ratio = 1.9, determined by XRF analysis) LDH, with a theoretical Zn/Al molar ratio of 2, was prepared by coprecipitation using NaOH as a buffer, following the process reported by Kovanda et al. (2010). ZnAl-1.9U-HT (Zn/Al molar ratio = 1.9, determined by XRF analysis) LDH, with a theoretical Zn/Al molar ratio of 2, was prepared by coprecipitation, involving the hydrolysis of urea as described by Liu et al. (2014).

The resulting LDHs were calcined at 400°C to obtain the corresponding mixed oxides (ZnAl-X-400, ZnAl-1.9K-400, and ZnAl-1.9U-400). It has to be stressed that the temperature 400°C is sufficient to the transformation of hydrotalcite to appropriate mixed oxide. The thermal treatment at 400°C have been chosen based on our previous work (Smolakova et al., 2018). Reconstructed materials (ZnAl-X-REH, ZnAl-1.9K-REH, and ZnAl-1.9U-REH) were prepared by stirring the mixed oxides in distilled water at a ratio of 1 g/50 ml at 25°C for 30 min. Subsequently, the suspension was filtered, and the residue was dried in a muffle oven for 1 h at 50°C under a N_2_ flow (100 ml/min).

### Characterisation of ZnAl Materials

X-ray diffraction (XRD) patterns were recorded using a diffractometer (MiniFlex 600, Rigaku, Japan) with a PDF-2 database and D/teX Ultra detector. The X-ray source was a CuKα tube operated at 40 kV and 15 mA. The slit width was set at 10 nm. The samples were measured at 10°/min at a step size of 0.02° and a 2θ range of 5–80°. The content of each phase was determined using reference intensity ratio (RIR) method. For the ZnAl LDHs and ZnAl RE-LDHs, the width of the space between the LDH layers was calculated as follows: interlayer width (Å) = $\frac{\text{c}}{3}-4.8$ (Zeng et al., 2017; Smolakova et al., 2018), where 4.8 Å is the width of the ZnAl layer (Sakr et al., 2013).

XRF analysis was used to determine the real Zn/Al molar ratio in the ZnAl LDHs. The analysis was conducted using a spectrometer (ARL 9400 XP) equipped with a rhodium lamp.

The specific surface area of the ZnAl materials was determined by N_2_ adsorption at 77 K in a static volumetric adsorption system (TriFlex analyzer, Micromeritics, Norcross, USA). The resulting adsorption isotherm was applied to Brunauer–Emmett–Teller functions to calculate the specific surface area of the materials.

*Ex-situ* diffuse reflectance ultraviolet–visible (DR UV–VIS) spectroscopy was performed using a GBC Cintra 303 spectrometer (GBC Scientific Equipment, Australia), equipped with an integrating sphere.

*In-situ* DR UV–VIS spectroscopy was performed using a spectrophotometer (Evolution 300, Thermo Scientific) equipped with a DR accessory containing a reaction chamber (The Praying Mantis, Harrick) (Smolakova et al., 2018; Dubnová et al., 2021). The DR spectra of the corresponding ZnAl LDHs were recorded in a He flow at temperatures ranging from 25 to 400°C; the temperature was constant for 4 h, after which the temperature was decreased to 50°C, and the rehydration of materials was conducted using home-made equipment.

Temperature-programmed (TP) techniques were measured on an Autochem II 2920 analyser (Micromeritics, Germany) connected to a mass spectrometer (Omnistar GSD 320, Pfeiffer Vacuum, Germany) with a quadrupole analyser and photomultiplier.

For the ZnAl LDHs and ZnAl RE-LDHs, TP analysis (TPA) was performed when the thermally controlled analysis programme was commenced, and the sample was heated in He atmosphere (25 ml/min) at temperatures ranging from 25 to 900°C at a heating rate of 10°C/min.

The TP desorption (TPD)-CO_2_ programme for CO_2_ adsorption on ZnAl hydrotalcites and ZnAl RE-LDHs commenced with pre-treatment at 50°C in He (25 ml/min) for 30 min. The duration was selected according to the temperature used to dry materials after rehydration. The mixture was cooled to 25°C, saturated with CO_2_ (10% CO_2_ in He atmosphere at 15 ml/min) for 30 min, and purged with He (25 ml/min), to remove physisorption molecules. Finally, desorption was performed at a heating rate of 10°C/min at a temperature range of 25–900°C.

The TPD-CO_2_ of the ZnAl mixed oxides was conducted as previously mentioned (Smolakova et al., 2018; Dubnová et al., 2021).

Inductively coupled plasma optical emission spectroscopy (ICP-OES) was used to determine the quantities of Na^+^, which were measured on an Agilent 5100 ICP-OES spectrometer.

### Catalytic Tests

The aldol condensation of furfural with acetone was performed in a glass-stirred batch reactor at 50°C. A mixture of acetone (Penta, p. a.) and furfural (Penta, p. a.), at a molar ratio of 1/10 pre-warmed to 50°C, was used. The rehydration of the freshly calcined mixed oxide was performed in water. After filtration, the RE-LDH catalyst was immediately transferred to the reactor and was studied throughout the aldol condensation for 4 h. Samples were retrieved from the reaction mixture during the experiment after 5, 10, 20, 40, 60, 90, 120, 150, 180, and 240 min. After filtration, the reaction products were analysed using an Agilent 7890A gas chromatograph equipped with a flame ionisation detector using an HP 5 capillary column to remove any residual catalyst. FAc-OH (4-(2-furyl)-4-hydroxybutan-2-one), FAc (4-(2-furyl)-3-buten-2-one), and F2Ac (1,4-pentadien-3-one-1,5-di-2-furanyl) were the main products formed. Acetone self-condensation products were observed to be insignificant. The carbon balance exceeded 97% in all experiments.

## Results and Discussion

### Structural Properties of ZnAl-Reconstructed LDHs

**FIGURE 1:**
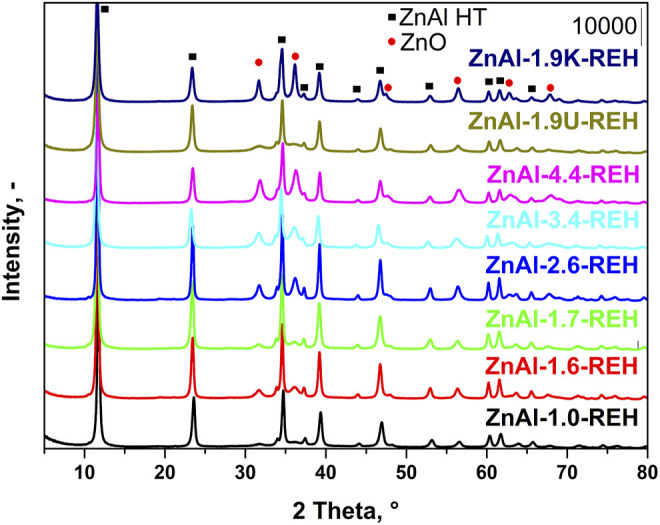
XRD patterns of reconstructed LDHs: ZnAl-X-REH with Zn/Al molar ratio 1.0–4.4, ZnAl-1.9K-REH and ZnAl-1.9U-REH.

Figure 1 shows the XRD patterns of RE-LDHs (ZnAl-X-REH with the Zn/Al molar ratios of 1.0–4.4, ZnAl-1.9K-REH, and ZnAl-1.9U-REH). The diffractograms of all materials contained diffraction lines, which are a characteristic of the presence of a crystalline LDH structure (2θ ≈ 11.6°, 23.5°, 34.6°, 39.2°, 46.8°, 52.9°, 60.2°, 61.6°, and 65.6°; reflections with planes (003), (006), (112), (015), (018), (1010), (110), (113), and (116); PDF 2, 01-080-6503 Quality: I) and a well-crystalline ZnO phase (2θ ≈ 31.8°, 36.2°, 56.5°, 62.9°, and 67.9°; reflections with planes (100), (101), (110), (103), (112); PDF 2, 01-080-6503 Quality: I).

Notably, the ZnAl RE-LDHs possessed crystalline LDH and ZnO structures. This is a fundamental difference compared to the corresponding LDHs containing either crystalline LDH or ZnO structures (ZnAl-1.9K-REH) or a pure crystalline LDH structure (ZnAl-X-HT with a Zn/Al molar ratio of 1.0–4.4 and ZnAl-1.9U-HT). The details are shown in the XRD patterns of the corresponding ZnAl LDHs (Supplementary Figure S1 **,** Supplementary Table S1) and ZnAl mixed oxides of the corresponding materials (Supplementary Figure S1, Supplementary Table S2) or our previous studies on ZnAl mixed oxides (Smolakova et al., 2018; Dubnová et al., 2021).

**FIGURE 2:**
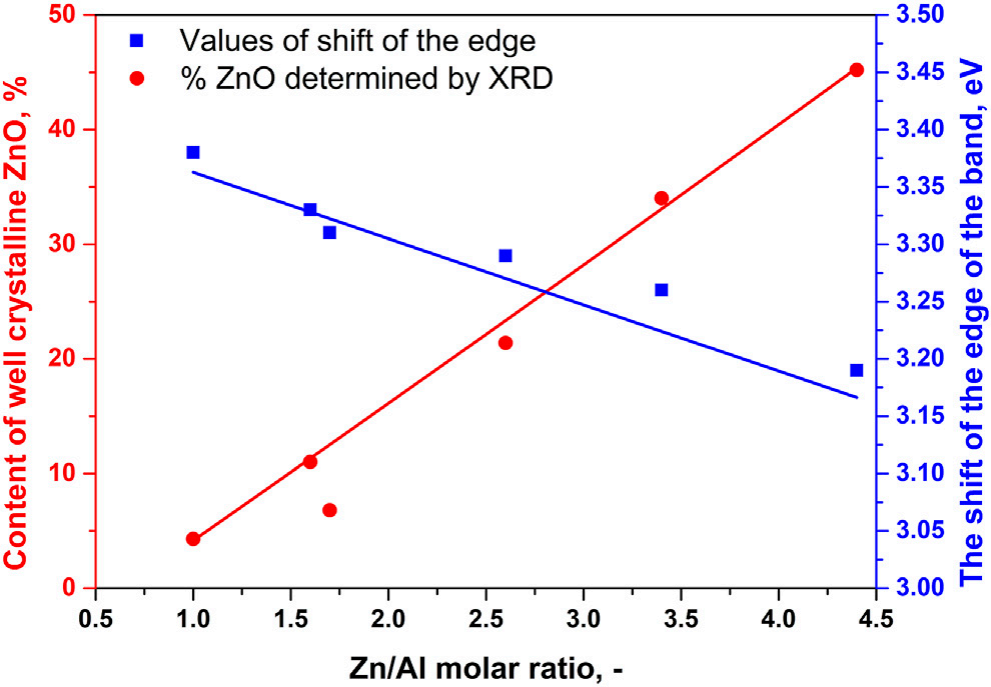
Dependences of the content of well crystalline ZnO phase determined by XRD (red marks, left side) and the shift of the edge of the band (blue marks, right side) on Zn/Al molar ration of ZnAl-X-REH reconstructed materials.

The content of well-crystalline ZnO phase (Table 1) in the RE-LDHs depended on the synthesis of the LDHs and Zn/Al molar ratio. Firstly, for the RE-LDHs with approximately the same ZnAl molar ratio, the highest ZnO content was observed for ZnAl-1.9K-REH (31.4%), followed by ZnAl-1.6-REH (11.0%), and ZnAl-1.9U-REH (5.9%) (Table 1). Secondly, the ZnO content in the RE-LDHs increased with an increasing Zn/Al molar ratio (ZnAl-X-REHs) from 3.6% for ZnAl-1.0-REH to 45.2% for ZnAl-4.4-REH (Figure 2).

**TABLE 1:** Crystallite size, lattice parameters, phase analysis and the shift of the edge of band from *ex-situ* DRS measurement of ZnAl-X-REH, ZnAl-1.9U-REH and ZnAl-1.9K-REH RE-LDHs.

Material	ZnO phase				Hydrotalcite phase				DRS
	wt%	D, Å	a, Å	c, Å	D, Å	a, Å	c, Å	x, Å	Eg, eV
ZnAl-1.0-REH	4.3	73	3.2638	5.2538	224	3.0592	22.5352	2.71	3.38
ZnAl-1.6-REH	11.0	50	3.2332	5.1668	103	3.0670	22.6663	2.76	3.33
ZnAl-1.7-REH	6.8	71	3.2448	5.1618	197	3.0677	22.6736	2.76	3.31
ZnAl-2.6-REH	21.4	78	3.2575	5.1729	154	3.0675	22.6136	2.74	3.29
ZnAl-3.4-REH	34.0	59	3.2621	5.2048	161	3.0829	22.9928	2.86	3.26
ZnAl-4.4-REH	45.2	83	3.2416	5.2277	212	3.0662	22.6047	2.74	3.19
ZnAl-1.9U-REH	5.9	42	3.2785	5.1994	114	3.0688	22.8225	2.81	3.61
ZnAl-1.9K-REH	31.4	84	3.2669	5.2320	191	3.0781	22.8989	2.83	3.22

*x is width of interlayer.

Table 1 lists the lattice parameters of the LDH phase in the RE-LDHs. The calculated width of the space between the layers in the RE-LDHs ranged from 2.71 to 2.86 Å. This corresponded to the presence of CO_3_^2−^ ions (2.7 Å) (Marcus, 2012) in the interlayer. The space between the layers in the corresponding original ZnAl LDHs ranged from 2.80 to 2.88 Å (Supplementary Table S1).

**FIGURE 3:**
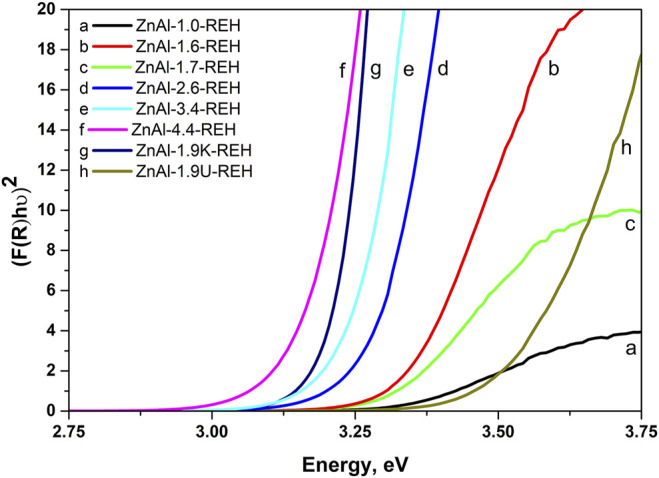
Recalculated DR spectra of ZnAl-X-REH reconstructed materials, ZnAl-1.9U-REH and ZnAl-1.9K-REH.

Figure 3 shows the DR spectra of the ZnAl RE-LDHs. DR spectroscopy (DRS) is suitable for determining the band-gap energy of ZnO semiconductors. Generally, the presence of ZnO semiconductors is the characteristic of a band-gap energy of 3.37 eV (Zhao et al., 2010; Ahmed et al., 2012). Wan et al. obtained a value of 3.05 eV for ZnO nanorods vertically aligned on the two LDH sides (Wan et al., 2012). For the ZnAl RE-LDHs, we specified the term “the shift of the edge of the band,” using the Kubelka–Munk function and Tauc plot (Table 1) (Smolakova et al., 2018). The advantage of “the shift of the edge of the band” is that it reflects the presence of crystalline and amorphous ZnO phases. This method has been used for ZnAl mixed oxides (Dubnová et al., 2021).

**FIGURE 4:**
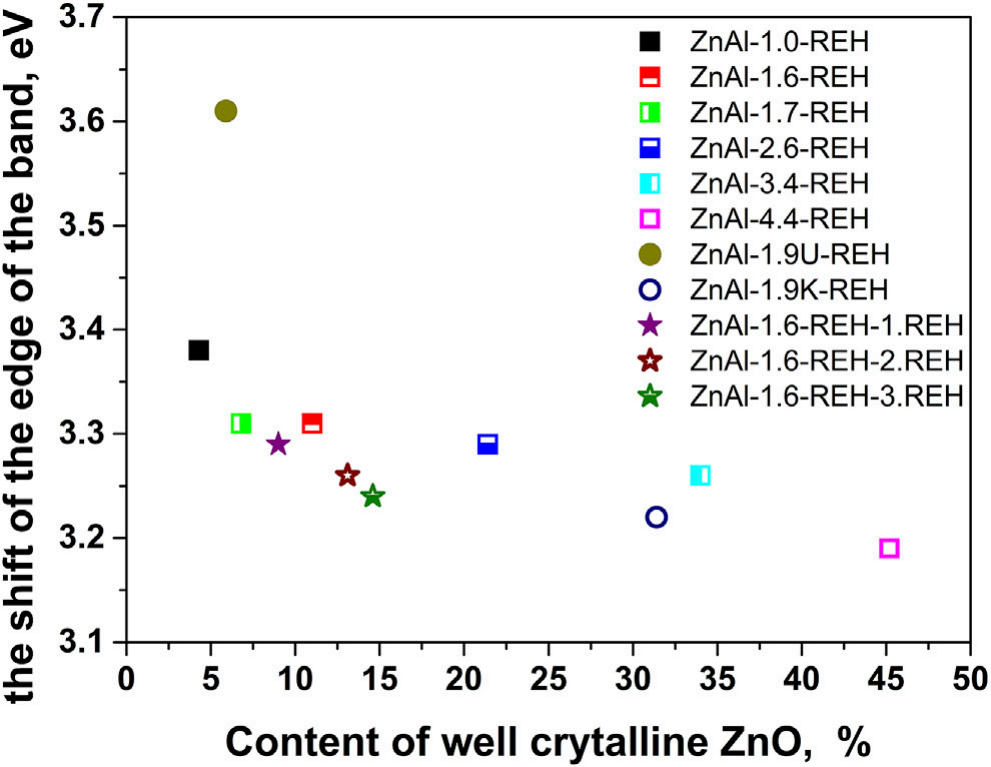
The dependence of the shift of the edge of band on the content of well crystalline ZnO phase determined by phase analysis from XRD.

Figure 4 shows the linear dependence of “the shift of the edge of the band” (DRS) for ZnAl-X-REH and ZnAl-1.9K-REH on the content of the well-crystalline ZnO phase (XRD). The only exception is that ZnAl-1.9U-REH possesses a “shift of the edge of the band” value above its observed linear dependence on the content of the well-crystalline ZnO for ZnAl-X-REH. This can be explained by the low contribution of the amorphous ZnO phase and/or the high degree of the interaction of the ZnO and LDH phases, resulting in the detection of a low content of the XRD-detectable ZnO phase in ZnAl-1.9U-REH (coprecipitation by hydrolysing urea) than in ZnAl-X-REH (coprecipitation using Na_2_CO_3_ as a buffer).

In addition, a comparison of the materials with approximately the same Zn/Al molar ratio showed that ZnAl-1.9U-REH contained the lowest content of the well-crystalline ZnO phase (5.9%), but laying on the observed linear dependence on the content of well-crystalline ZnO for ZnAl-X-REH. It was suggested that at the same Zn/Al molar ratio, the coprecipitation by hydrolysing urea resulted in a ZnAl-1.9U-REH material with a greater degree of interactions between the ZnO and LDH phases than in ZnAl-1.6-REH and ZnAl-1.9K-REH.

While in the case of LDH it is possible to prepare pure ZnAl LDHs with any detectable ZnO phase, ZnO phase was always observed in ZnAl RE-LDHs. In agreement, the presence of ZnO phase in RE-LDHs have also been reported by other authors (Tajuddin et al., 2019). Tajuddin et al. observed increasing intensity ratio of ZnAl LDH phase to ZnO phase in hydrothermally treated ZnAl RE-LDHs with an increasing ZnAl molar ratio from 41,2% (Zn/Al = 1.6) to 82,2% (Zn/Al = 3.3). Thus, there was not reported complete reconstruction of hydrothermally treated ZnAl LDHs. In contrast to that, we obtained the highest level of the LDH phase reconstruction for ZnAl-1.0-REH with a low Zn/Al molar ratio value, i.e. for ZnAl-1.0-REH containing 4.3 wt% ZnO phase and ZnAl-1.9U-REH containing 5.9 wt% ZnO phase (both materials without any detectable ZnO phase in ZnAl LDHs).

### Basic Properties of ZnAl-Reconstructed LDHs

**FIGURE 5:**
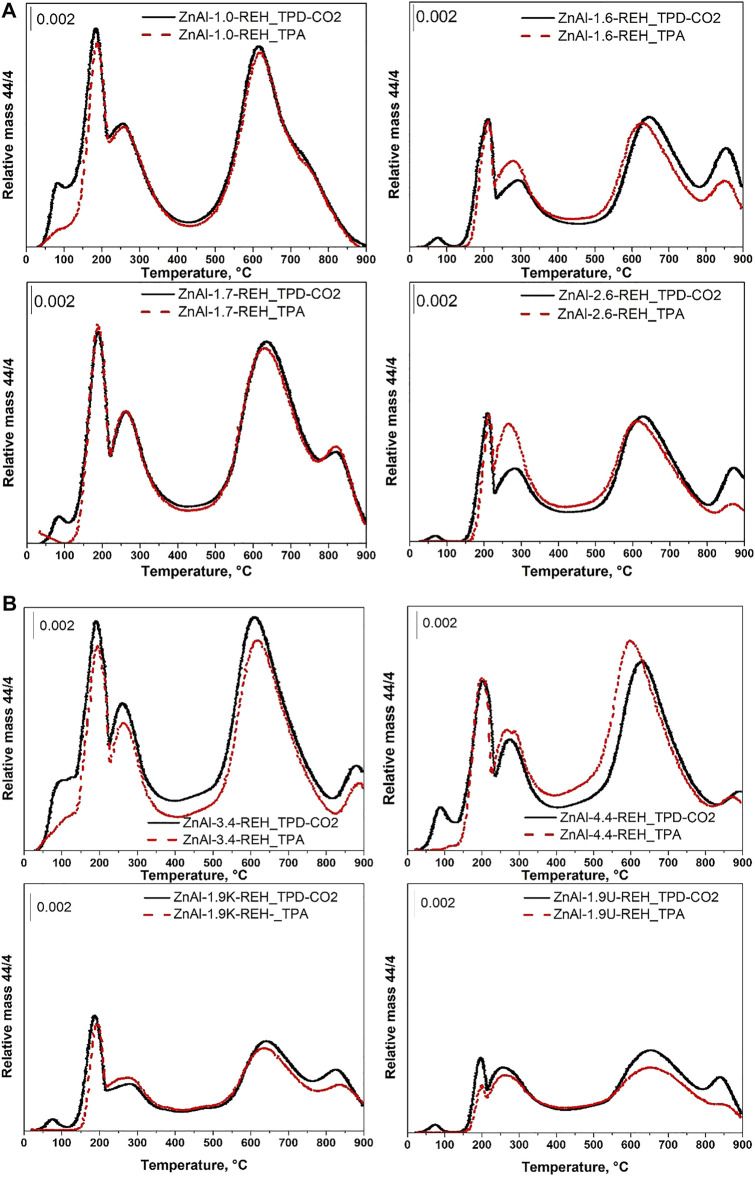
**(A)** TPD-CO_2_ and TPA (experiment without CO_2_ adsorption) profiles of ZnAl RE-LDHs: ZnAl-1.0-REH, ZnAl-1.6-REH, ZnAl-1.7-REH, ZnAl-2.6-REH. **(D)** TPD-CO_2_ and TPA (experiment without CO_2_ adsorption) profiles of ZnAl RE-LDHs: ZnAl-3.4-REH, ZnAl-4.4-REH, ZnAl-1.9K-REH, ZnAl-1.9U-REH.

Figure 5 shows the TPD-CO_2_ profiles of the ZnAl RE-LDHs. Desorption peaks were observed at maximum temperature ranges of 70–90, 180–210, and 260–280°C. Other desorption peaks were observed above 400°C (above the temperature of the thermal treatment performed during the synthesis of the materials). Above 400°C, desorption peaks show both desorption of CO_2_ from the strong basic sites that is usually reported up to 480°C and CO_2_ released from the residual carbonate species, which was not decomposed during calcination of the hydrotalcite-like precursor or that can be formed with interaction of RE-LDHs with air. In order to describe the interaction of ZnAl RE-LDHs with atmospheric CO_2_ and possible carbonate formation, we performed both TPA and TPD-CO_2_ experiments up to 900°C. Notably, with the rising temperature during the TPD-CO_2_, the release and subsequent detection of CO_2_ could be reflected from the amount of CO_2_ adsorbed on the materials during the TPD-CO_2_, and the release of CO_2_ present in the RE-LDH prior to CO_2_ adsorption occurred during the TPD-CO_2_. Therefore, for all ZnAl RE-LDHs, the CO_2_ signals during the TPD-CO_2_ and TPA (experiment without CO_2_ adsorption) were compared (Figure 5).

A desorption peak was at a maximum at a temperature range of 70–90°C and was observed only during the TPD-CO_2_ of ZnAl RE-LDHs but not during the TPA (Figure 5). This desorption peak represented the amount of CO_2_ adsorbed on the material during the TPD, indicating the presence of weak basic sites (Smolakova et al., 2018). The number of weak basic sites could not be unambiguously determined from the intensity of this CO_2_ desorption peak, as the peak was often affected by the presence of desorption peaks at temperatures higher than 90°C.

**FIGURE 6:**
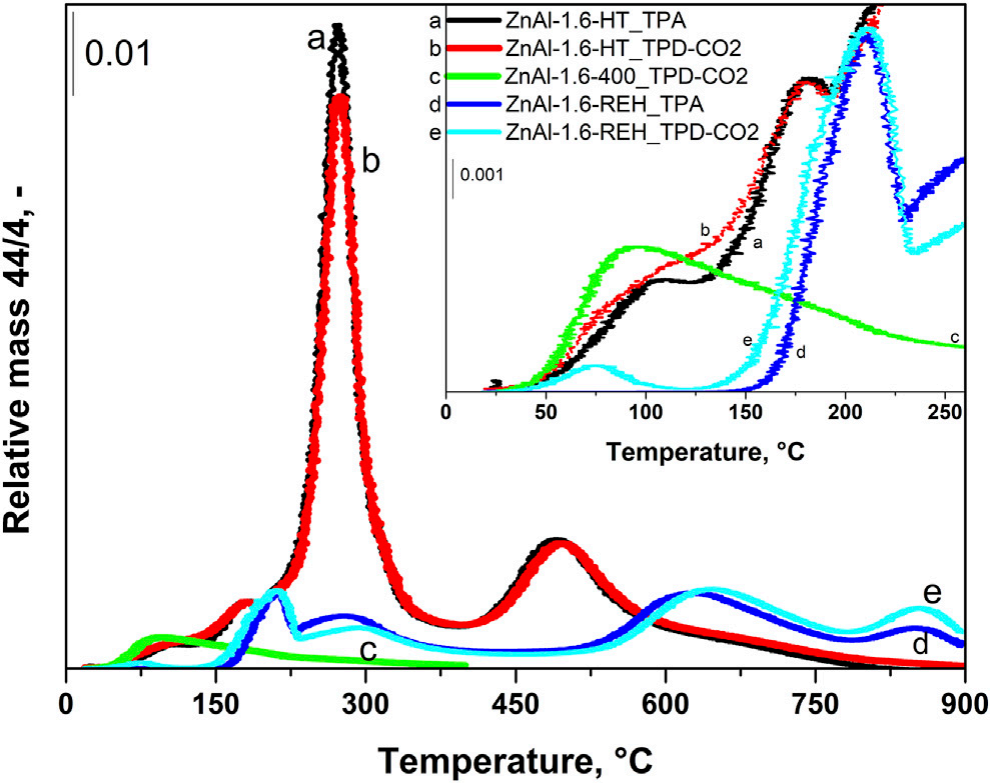
TPD-CO_2_ profiles of ZnAl-1.6-HT hydrotalcite, ZnAl-1.6-400 mixed oxide, ZnAl-1.6-REH reconstructed LDH and TPA (experiment without CO_2_ adsorption) profiles of ZnAl-1.6-HT hydrotalcite and ZnAl-1.6-REH RE-LDH.

The intensities of the CO_2_ desorption peaks were at a maximum within temperatures ranging from 180 to 210 and 260–280°C and were close in case of both the TPD-CO_2_ and TPA. Although a slight discrepancy could be observed for ZnAl-1.6-REH and ZnAl-2.6-REH (the TPA signal was relatively intense for the desorption peak at a maximum within the temperature range of 260–280°C) and ZnAl-1.9U-REH (the TPA signal was similarly intense but within a temperature range of 180–210°C). This would perhaps occur in exceptional cases rather than in systematic circumstances. This is perhaps because the desorption peaks with maxima within the temperature ranges 180–210 and 260–280°C originated from the rapid interaction of atmospheric CO_2_ with the RE-LDHs, which rapidly occurred when the RE-LDHs came in contact with air. Thus, these desorption peaks were observed for the TPD-CO_2_ and TPA. Noteworthily, these desorption peaks were not observed for the ZnAl mixed oxides (Figure 6); however, they were close to those present for the TPD-CO_2_ and TPA signals of the corresponding original ZnAl LDHs (Figure 6). The presence of CO_2_ desorption peaks with maxima within the temperature ranges of 180–210 and 260–280°C, during the TPD-CO_2_ and TPA, could be explained by the loss of physically adsorbed CO_2_ and the decomposition of the interlayer CO_3_^2−^ (Cavani et al., 1991; Tu et al., 1999; Zhao et al., 2010). This indicated that although the OH^−^ ions could be present in the interlayer of the RE-LDH (Marcus, 2012), these ions could easily be replaced by more thermodynamically stable CO_3_^2−^ ions.

Figure 6 compares the desorption curves of the selected ZnAl-1.6 LDH, corresponding mixed oxides, and RE-LDH during the TPD-CO_2_ and TPA (the rest of the materials can be found in Supplementary Figure S2). In the TPD-CO_2_ (red) and TPA (black) of ZnAl-1.6-HT LDH, CO_2_ desorption peaks with maxima at temperatures of 105, 180, and 270°C were observed. The intensity of the desorption peak at 105°C was slightly higher during the TPD-CO_2_ than during the TPA. For the other cases, the intensities of the desorption peaks were the same. These CO_2_ desorption peaks corresponded to the loss of physically adsorbed CO_2_ and the decomposition of the interlayer CO_3_^2−^ groups. During the TPD-CO_2_ (green) of the ZnAl-1.6-400 mixed oxide, there was a dominant desorption peak with a maximum at 90°C associated with its shoulder at high temperatures. During the TPD-CO_2_ (light blue) and TPA (blue) of ZnAl-1.6-REH, CO_2_ desorption peaks were observed with maxima at 75, 210, and 280°C (Figure 6). In addition, the desorption peaks with maxima at 210 and 280°C observed during the TPD-CO_2_, and the TPA experiments of ZnAl-1.6-REH were observed at higher temperatures than in experiments with ZnAl-1.6-HT, where the maxima of the desorption peaks were observed at 180 and 270°C. This shows the reorganisation (reintroduction) of the LDH structure in the RE-LDHs as compared to the LDHs.

### What Occurs During the Repeated Transformation of ZnAl Mixed Oxides Into RE-LDHs?

The ZnAl-LDH was repeatedly reconstructed with intermediate calcination (three times) to clarify as to which phenomenon occurred during the repeated transformation of ZnAl mixed oxides into RE-ZnAl-LDHs. Simultaneously, the change in the structural (*in-situ* DRS and XRD) and basic properties (TPD-CO_2_, including the comparison with TPA) was measured.

**FIGURE 7:**
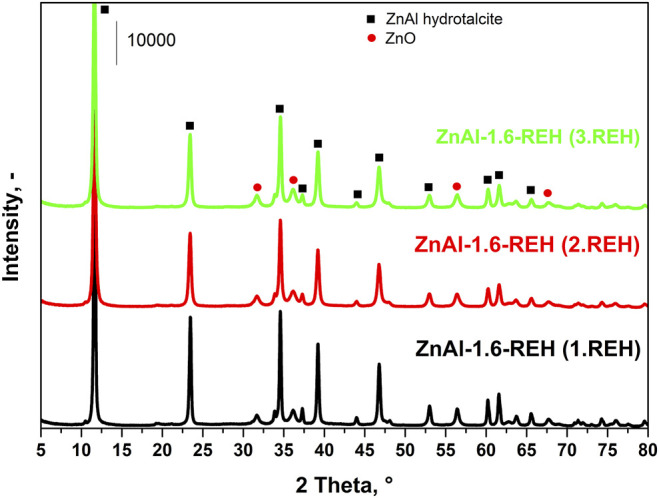
XRD patterns of three subsequent cycles of reconstruction of ZnAl-1.6-400 mixed oxide.

Figure 7 shows the XRD patterns of ZnAl-1.6-RE-LDHs after the first, second, and third cycles of the reconstruction of the LDH structure in the liquid phase. The diffractograms of the materials contained diffraction lines, which are a characteristic of the presence of a crystalline LDH structure and a well-crystalline ZnO phase. The repeated reconstruction of the LDH structure led to an increase in the content of well-crystalline ZnO, which possessed a small crystallite (D) and an increased width of the space between the layers (Table 2). However, the increasing number of cycles of the reconstruction of the LDH structure did not affect any amorphous ZnO phase following “the shift of the edge of the band” (Table 2), which corresponded to the aforementioned linear dependence of “the shift of the edge of the band” on the content of the well-crystalline ZnO (Figure 4).

**TABLE 2:** Crystallite size, lattice parameters, phase analysis and the shift of the edge of band from *ex-situ* and *in-situ* DRS measurement of three following circle of reconstruction of ZnAl-1.6-REH material.

ZnAl hydrotalcite	ZnO phase				Hydrotalcite phase				DRS *ex-situ*	DRS *in-situ*
	wt%	D, Å	a, Å	c, Å	D, Å	a, Å	c, Å	x, Å	E_g_, eV	E_g_, eV
ZnAl-1.6-REH (1.REH)	9	107	3.2493	5.1713	316	3.0675	22.6276	2.74	3.29	3.77
ZnAl-1.6-REH (2.REH)	13.1	105	3.2534	5.3133	265	3.0656	22.6591	2.75	3.26	3.31
ZnAl-1.6-REH (3.REH)	14.6	85	3.2774	5.2008	263	3.0754	22.8954	2.83	3.24	3.29

**FIGURE 8:**
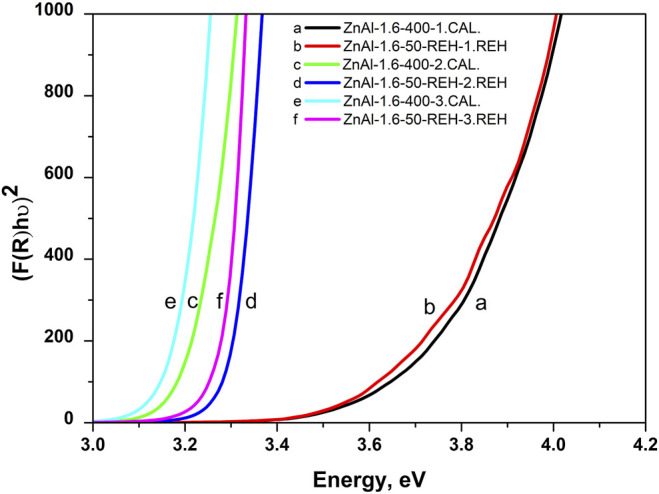
*In-situ* DRS during repeating transformation of mixed oxide into reconstructed materiel, i.e. the spectra of ZnAl-1.6-400 mixed oxide (after thermal treatment of ZnAl-1.6-HT at 400°C for 4 h), ZnAl-1.6-50-REH (after gas phase treatment of ZnAl-1.6-400 mixed oxide at 50°C for 4 h), ZnAl-1.6-400 mixed oxide after subsequent second calcination, ZnAl-1.6-50-REH after subsequent second gas phase treatment, ZnAl-1.6-400 mixed oxide after subsequent third calcination and ZnAl-1.6-50-REH after subsequent third gas phase treatment.

Figure 8 shows the *in-situ* DRS results during the transformation of ZnAl-1.6-HT into a mixed oxide (thermal treatment), the subsequent transformation of the mixed oxide into ZnAl-1.6-REH (treatment in the gas phase at 50°C), and ZnAl-1.6-REH to mixed oxide (three times). Firstly, the value of ‘the shift of the edge of the band’ decreased with an increase in the number of cycles for the reconstruction of LDH structure (3.77, 3.31, and 3.29 eV). This trend correlated with that in the *ex-situ* DRS; however, the observed values of “the shift of the edge of the band” were relatively high after the first cycle, 3.77 eV (gas phase) vs. 3.29 eV (liquid phase) (Table 2). This is because the *in-situ* gas phase reconstruction after the first cycle led to a low LDH structure recovery than in the liquid phase. Conversely, the values of “the shift of the edge of the band” were comparable after the second and third reconstruction cycles in the liquid and gas phases (*ex-situ* spectra: 3.24–3.31 eV, Table 2). Thus, a repeated or sufficiently long reconstruction in the gas phase can result in a similar LDH structure recovery as in the liquid phase. Secondly, the value of “the shift of the edge of the band” increased during the thermal treatment of RE-LDH into mixed oxide (Figure 8), confirming that a greater interaction of ZnO and Al_2_O_3_ oxides was observed for the ZnAl mixed oxide than for the ZnO and LDH phases in ZnAl RE-LDHs.

**FIGURE 9:**
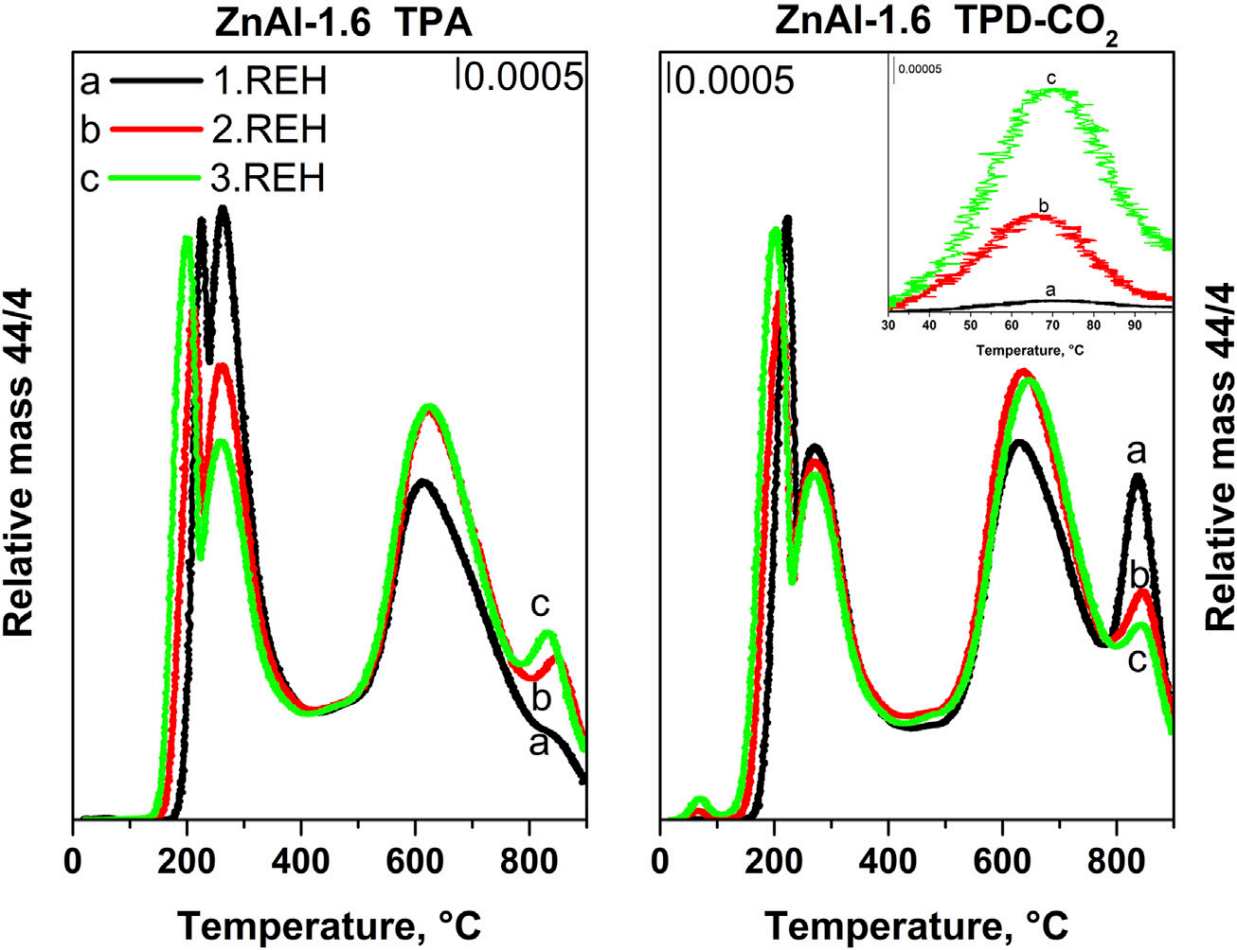
TPA (experiment without CO_2_ adsorption) and TPD-CO_2_ profiles of ZnAl-1.6 RE-LDHs during repeating transformation of mixed oxide into reconstructed material: 1. REH – first rehydration circle, 2. REH – second rehydration circle, 3. REH – third rehydration circle.

Figure 9 shows the TPD-CO_2_ of ZnAl-1.6-REH after the first, second, and third cycles of the LDH structure reconstruction. First, the maxima and the intensities of CO_2_ desorption peaks at 225 and 260°C (ZnAl-1.6-REH after the first cycle) shifted to low temperatures with an increase in the number of cycles. This showed that the degree of interaction of the interlayer CO_3_^2−^ groups with the LDH structure decreased with an increase in the number of cycles. In addition, the space between the layers increased with an increase in the number of LDH structure reconstruction cycles, which perhaps weakened the fixation of the CO_3_^2−^ groups. Secondly, the intensity of the CO_2_ desorption peak with a maximum in the temperature range of 65–70°C represented an increase in the number of weak Brønsted basic sites with an increasing number of reconstruction cycles. Thus, although the presence of OH^−^ ions in the interlayer was not confirmed, slight changes in the width of the interlayer between the ZnAl LDHs and ZnAl RE-LDHs suggested that the distribution of ions in the interlayer had changed.

### The Risk of Na Leaching

In principle, RE-LDHs can be prepared either by mixing the composite oxide with decarboxylated water in the liquid phase, by rehydrating the mixed oxide in an inert gas stream of decarbonated water vapour, or by adding water directly in the reaction mixture. In order the analyse the risk of Na leaching, during the reconstruction of ZnAl MOs, we have focused on the individual steps of the synthesis of ZnAl-1.6-HT, ZnAl-1.9K-HT, and ZnAl-1.9U-HT LDHs and their subsequent modifications to the corresponding mixed oxides and RE-LDHs (ICP-OES).

Table 3 lists the pH values of the filtrates during the washing of the synthesised LDHs with water. For ZnAl-1.6-LDH (using Na_2_CO_3_ as a buffer), we used a high amount of distilled water, 2.5 L per Gram of LDH, to achieve a neutral pH for the filtrate. Thereafter, ZnAl-1.6-LDH was carefully washed to remove all residual Na-species and at the end of filtration, marginal Na was leached from ZnAl-1.6-LDH into the filtrate, and the pH value of the filtrate was practically constant. There was analysed total amount of Na in the last 10 L of the filtrate and after recalculation. When converting the Na content in the last 10 L of filtrate to the equivalent amount of Na per Gram of solid material, the amount of Na in the last 10 L of the filtrate was equal to 2.3 mg of Na per Gram of the solid ZnAl-1.6-LDH. Despite the large amount of water used during the filtration of ZnAl-1.6-LDH and the low amount of Na present in the filtrate at the end of the filtration, the chemical analysis of the solid ZnAl-1.6-400 mixed oxide confirmed the presence of a relatively high amount of Na, i.e., 18.0 mg Na per Gram of ZnAl-1.6-400 mixed oxide.

**TABLE 3:** Content of Na and pH value during the preparation of ZnAl hydrotalcites, ZnAl mixed oxides and ZnAl reconstructed material.

	ZnAl-1.6			ZnAl-1.9K			ZnAl-1.9U		
Filtration after using x l of water	pH	Na, c (mg/l)	mg Na/g_kat_	pH	Na, c (mg/l)	mg Na/g_kat_	pH	Na, c (mg/l)	mg Na/g_kat_
Distilled water	6.43	—	—	6.24	—	—	—	4.5	1.8
10 L water	10	—	—	9.51	—	—	—	—	—
20 L water	8.79	—	—	6.35	3.1	2.58	—	—	—
21 L water	—	—	—	**6.33**	**2**	**0.17**	—	—	—
30 L water	8.14	—	—	—	—	—	—	—	—
40 L water	7.74	—	—	—	—	—	—	—	—
50 L water	7.45	—	—	—	—	—	—	—	—
60 L water	**6.92**	**5**	**2.3**	—	—	—	—	—	—
ZnAl mixed oxide		48.7	18.0	—	4.1	1.64	—	4.5	1.8
ZnAl-REH filtrate after rehydration		256	14.00	—	—	—	—	—	—
ZnAl-REH powder sample		6.3	2.3	—	—	—	—	—	—

Subsequently, the ZnAl-1.6-400 mixed oxide was rehydrated in water, and the amount of Na present in ZnAl-1.6-REH and the aqueous phase was studied. While the solid ZnAl-1.6-REH powder contained 2.3 mg Na per Gram of the material, the aqueous phase contained 14.0 mg Na per Gram of the material (after recalculation of the Na content in the liquid phase to the amount of solid material). Thus, washing of ZnAl-1.6-400 mixed oxide by water led to very easy leaching of those Na that was not leached during the washing ZnAl-1.6-LDH. In summary, the starting ZnAl-1.6-400 mixed oxide contained 18.0 mg Na per Gram of the mixed oxide, of which 14.0 mg Na was released into the water during rehydration and 2.3 mg Na remained in the solid ZnAl-1.6 RE-LDH material. The difference in the Na balance (18.0 vs. 14.0 + 2.3) represented an experimental error connected with the determination of the amount of material and the recalculation of Na content in the liquid phase to the solid material. However, the results showed that Na leached from the solid ZnAl-1.6-400 mixed oxides into pure water during reconstruction. Thus, it can be concluded that the process of ZnAl mixed oxide reconstruction should be performed in pure water and that RE-LDH can be used in the reaction after filtration. The risk of Na leaching has not been reported, as it is typically checked during the synthesis of LDH and not thereafter. It should be stressed that this observation has been observed only in the case of ZnAl-based materials prepared by coprecipitation using an aqueous Na_2_CO_3_ solution as a buffer.

NaOH was used as a precipitant and buffer for the synthesis of ZnAl-1.9K-HT LDH. Contrary to ZnAl-1.6-HT, the amount of NaOH required was lesser than that of Na_2_CO_3_, to achieve a certain pH during the synthesis of LDH. Thus, a relatively low quantity of Na was present in the resulting ZnAl-1.9K-HT than in the ZnAl-1.6-HT, which attributed to the small amount of water required to wash the resulting ZnAl-1.9K. Consequently, 1.75 L of distilled water per Gram of LDH was required to wash approximately 12 g of ZnAl-1.9K-HT. The Na content of the last 1 L of the filtrate was determined and converted to the total amount of Na released from 1 g of LDH using 1 L of water to yield 0.17 mg Na per Gram LDH. The resulting ZnAl-1.9K-400 mixed oxide contained an extremely low Na content, namely 1.64 mg Na per Gram mixed oxide.

Na-containing chemicals were not used in the synthesis of ZnAl-1.9U. Thus, this material did not represent a risk in terms of Na leaching from a solid catalyst.

### Performance of ZnAl RE-LDHs in the Aldol Condensation of Furfural

**FIGURE 10:**
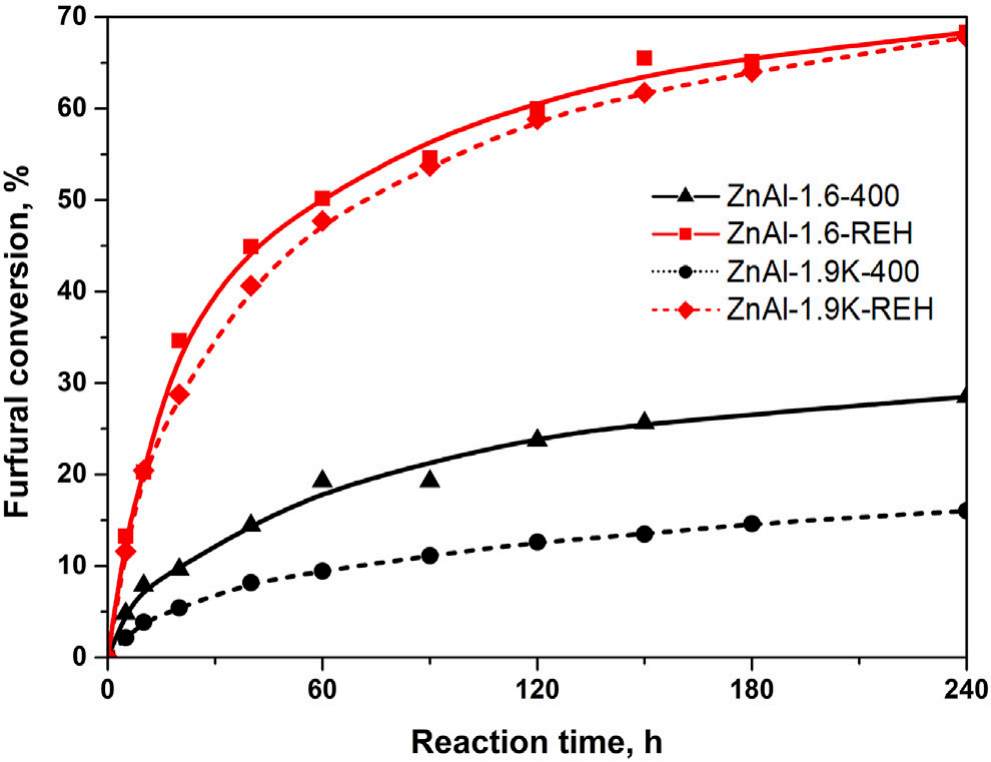
Conversion of furfural in aldol condensation reaction using ZnAl mixed oxides and ZnAl reconstructed LDHs catalysts.

Figure 10 shows the furfural conversion observed for representative RE-LDHs (ZnAl-1.9K-REH and ZnAl-1.6-REH) in comparison to their corresponding mixed oxides (ZnAl-1.9K-400 and ZnAl-1.9K-400). Both the reconstructed materials exhibited significantly higher furfural conversion than their corresponding mixed oxides. Notably, we previously reported the catalytic behaviour of ZnAl mixed oxides; therefore, we did not focus on this topic (Smolakova et al., 2018; Dubnová et al., 2021). In contrast to the previous study, we have used different reaction conditions that were optimised for RE-LDHs (1 g of the catalyst instead of 2 g).

**FIGURE 11:**
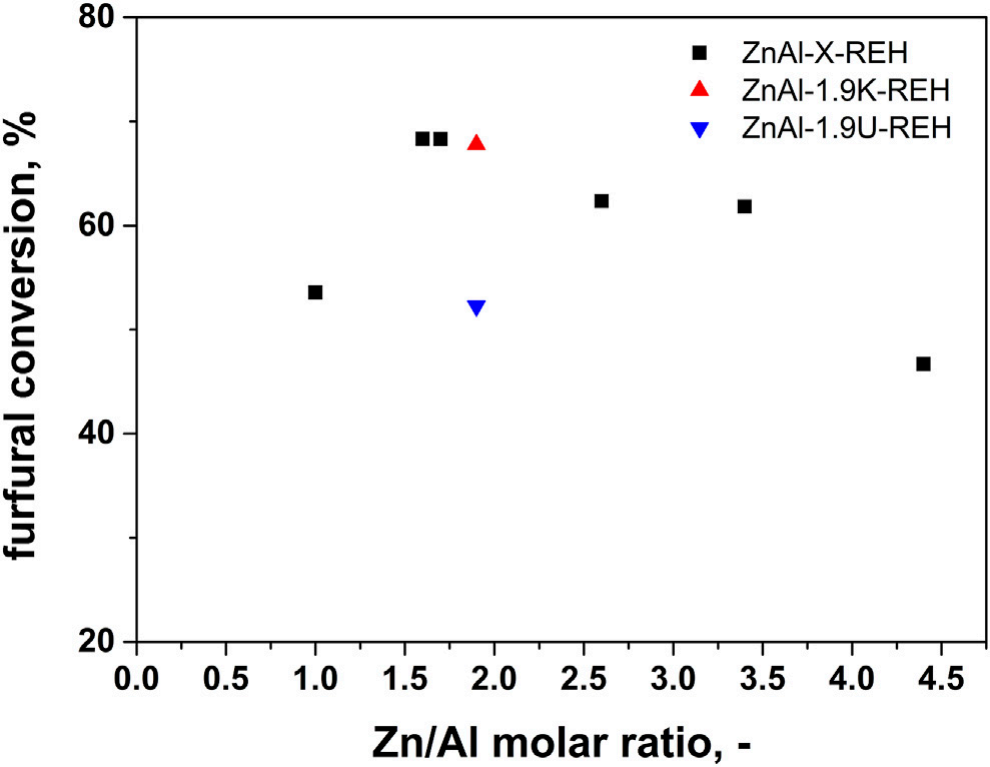
Dependence of furfural conversion on Zn/Al molar ratio in aldol condensation reaction using ZnAl reconstructed LDHs catalysts.

Figure 11 shows the catalytic performance of ZnAl-X-REH RE-LDHs in the aldol condensation of furfural. The highest conversion of furfural was observed for ZnAl-1.6-REH (68% after 360 min). Comparing the furfural conversions of the reconstructed materials originating from three LDHs, the furfural conversion increased in the order ZnAl-1.6-REH ≈ ZnAl-1.9K-REH > ZnAl-1.9U-REH. This order was different from that for the mixed oxides, where we reported the highest furfural conversion for ZnAl-1.9U-400, compared to those for ZnAl-1.6-400 and ZnAl-1.9K-400. Although the reconstructed materials possessed high furfural conversions, no clear correlation was observed between the furfural conversion and shift in band-gap energy, surface area, and ZnO content. This showed that the catalytic performance of ZnAl-RE-LDHs was complex and dependent on several parameters. Many authors previously emphasised the role of basic sites during furfural conversion. However, the amount and the distribution of the basic sites could not be determined for the reconstructed materials because of the overlapping of the desorption peak reflecting the basicity of the reconstructed materials and the desorption peaks originating from the carbonate species present in the RE-LDHs.

It is hard to make a deeper comparison with other groups as the individual works differ in the reaction conditions (amount of catalyst, reaction composition, temperature). For example, there have been reported conversion of furfural 70% for MgZr mixed oxide (Sádaba et al., 2011) and between 80-100% for Mg-Al mixed oxides (Hora et al., 2015). It should be stress that ZnAl MOs possessed furfural conversion from 40% (Smoláková et al., 2017a) to 81% (Smolakova et al., 2018). A wide range of furfural conversion were observed depending on the synthesis of the material and the resulting amount of well-crystalline ZnO phase in ZnAl mixed oxides (Dubnová et al., 2021). In this manuscript, Figure 10 shows the significantly higher furfural conversion for ZnAl RE-LDHs than for their corresponding ZnAl MOs.

While more attention is focused to MgAl-based materials than to ZnAl-based materials in the literature, works showing the benefits of ZnAl-based materials have recently begun to mention. In addition, reconstructed MgAl and ZnAl LDHs brings new opportunities and differences between these two types of materials. ZnAl mixed oxide possessed higher ester yield in transesterification of rapeseed oil with methanol than MgAl mixed oxide (Smoláková et al., 2017b). High potential of ZnAl-based materials seems to be in the presence of redox sites (Rosset et al., 2021). There has been reported that ZnNiAl RE-LDHs with Zn showed higher crystallinity and reducibility, whereas the MgNiAl RE-LDHs showed higher basicity. It leads to higher CO_2_ conversion in dry reforming (Rosset et al., 2021). The addition of Zn was found more effective in keeping the Ni in its metallic state thereby enhancing its stability (Kumar and Pant, 2020). Thus, detail study describing the changes in the structural and basic properties of ZnAl MOs during their transformation into ZnAl RE-LDHs could be beneficial for many scientific groups.

## Conclusion

ZnAl LDHs were prepared using three methods (coprecipitation using Na_2_CO_3_ as a buffer, NaOH as a buffer, and hydrolysing urea), from which the corresponding mixed oxides and RE-LDHs were prepared. This study described the supposed memory effect in ZnAl RE-LDHs. The individual preparations fundamentally differed in the ZnAl properties of the RE-LDHs. The most fundamental differences were observed in the content of the well-crystalline ZnO phase, the ability to reconstruct the LDH structure, and the number of basic sites.

• All ZnAl RE-LDHs contained LDH and ZnO structures. The content of the well-crystalline ZnO phase depended on the synthesis and increased with an increase in the Zn/Al molar ratio. Its content was higher in ZnAl RE-LDHs than in the corresponding ZnAl LDHs, and it was present even in materials originating from ZnAl-LDH with a pure LDH structure.
• The content of the well-crystalline ZnO phase was lower in ZnAl RE-LDH originating from the coprecipitation method that involved the hydrolysis of urea, than that originating from the coprecipitation method using NaOH or Na_2_CO_3_ as buffers.
• The repeated reconstruction of the LDH structure led to an increase in the content of the well-crystalline ZnO phase that possessed a small crystallite, an increase in the width of the space between the layers, decrease in the interaction of the interlayer CO_3_^2−^ groups with the LDH structure, and an increase in the number of weak basic sites.
• Although the LDH structure recovery was slower in the gas phase than in the liquid phase, the prolonged time in the gas phase led to a similar LDH structure recovery to that in the liquid phase.

## Data Availability

The original contributions presented in the study are included in the article/Supplementary Material, further inquiries can be directed to the corresponding author.
