# Supplementary material for: Reconstruction of the ZnAl Mixed Oxides Into the Layered Double Hydroxide Catalysts Active in the Aldol Condensation of Furfural: The Role of ZnO Particles
Source: Front Chem. 2022 Jan 14;9:803764. doi: 10.3389/fchem.2021.803764 (PMC8795589; doi:10.3389/fchem.2021.803764)
Supplement: Supplementary file 1 [file DataSheet1.docx]

**
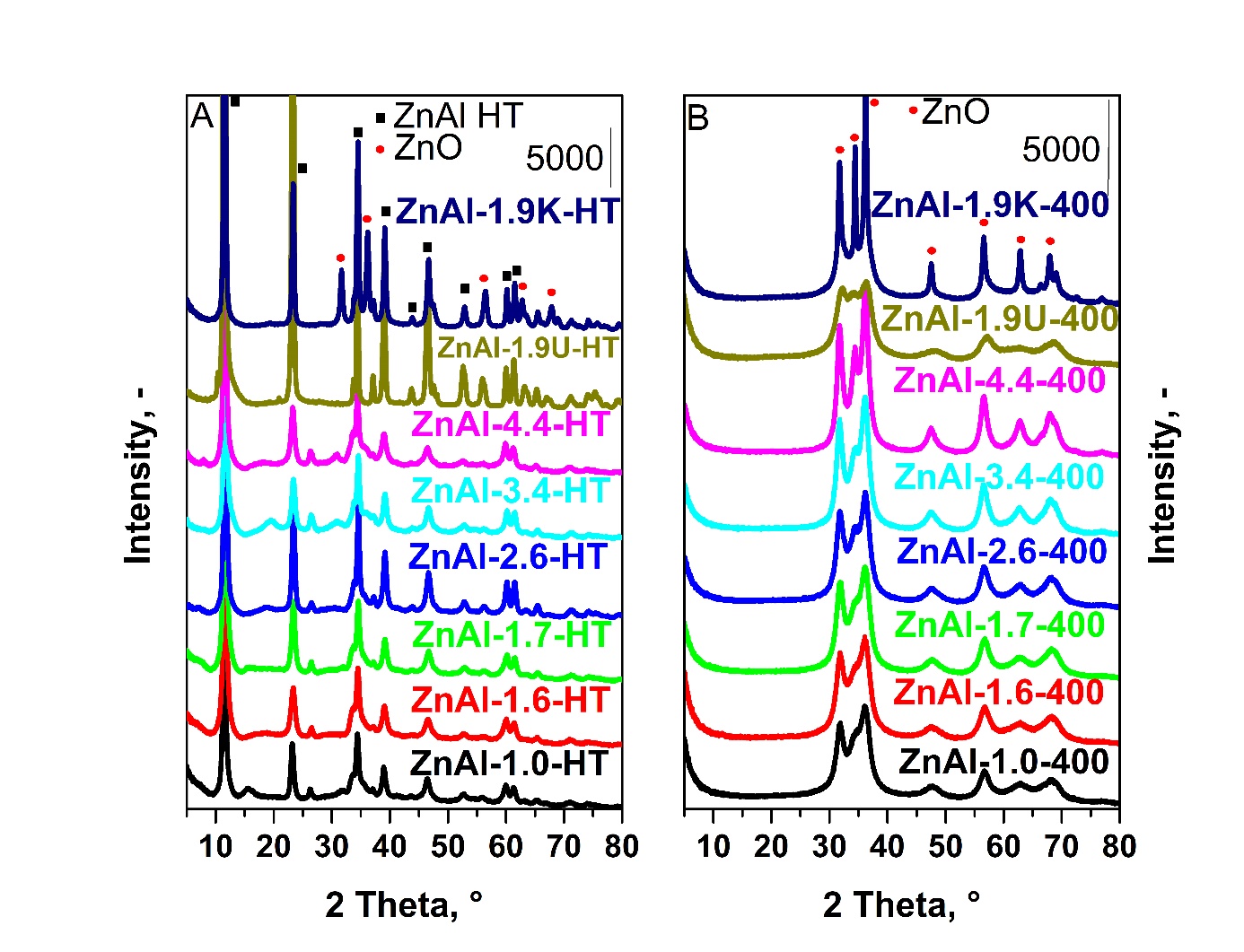
Fig. S1** XRD patterns of ZnAl LDHs (A): ZnAl-X-HT with Zn/Al molar ratio 1.0 - 4.4, ZnAl-1.9K-HT and ZnAl-1.9U-HT; and ZnAl mixed oxides (B): ZnAl-X-HT with Zn/Al molar ratio 1.0 - 4.4, ZnAl-1.9K-HT and ZnAl-1.9U-HT.

**Table S1** Lattice parameters, crystallite size, phase analysis, basal plane spacing and specific surface area of ZnAl LDHs.

| **ZnAl hydrotalcite** | **ZnO phase** | | | | **Hydrotalcite phase** | | | | **S_BET,_ m^2^/g** |
| --- | --- | --- | --- | --- | --- | --- | --- | --- | --- |
|  | **wt. %** | **D, Å** | **a, Å** | **c, Å** | **D, Å** | **a, Å** | **c, Å** | **x, Å** |  |
| ZnAl-1.0-HT | -- | - | - | - | 23 | 3.0843 | 22.9730 | 2.86 | 98 |
| ZnAl-1.6-HT | - | - | - | - | 83 | 3.0811 | 22.8552 | 2.82 | 78 |
| ZnAl-1.7-HT | - | - | - | - | 111 | 3.0748 | 22.8306 | 2.81 | 51 |
| ZnAl-1.8-HT | - | - | - | - | 46 | 3.0751 | 22.7998 | 2.80 | 42 |
| ZnAl-2.6-HT | - | - | - | - | 9 | 3.0749 | 22.7914 | 2.80 | 31 |
| ZnAl-3.4-HT | - | - | - | - | 92 | 3.0820 | 22.9215 | 2.84 | 50 |
| ZnAl-4.4-HT | - | - | - | - | 95 | 3.0916 | 23.0508 | 2.88 | - |
| ZnAl-1.9U-HT | ***-*** | ***-*** | ***-*** | **-** | 985 | 3.0845 | 23.0505 | 2.88 | 15 |
| ZnAl-1.9K-HT | 21.2 | 112 | 3.2666 | 5.2159 | 264 | 3.0795 | 22.9476 | 2.85 | 65 |

**Table S2** Lattice parameters, crystallite size, phase analysis and specific surface area of ZnAl mixed oxides.

| **ZnAl mixed oxide** | **ZnO phase** | | | | **S_BET,_ m^2^/g** |
| --- | --- | --- | --- | --- | --- |
|  | **wt. %** | **D, Å** | **a, Å** | **c, Å** |  |
| ZnAl-1.0-400 | 100 | 35 | 3.2415 | 5.2345 | 46 |
| ZnAl-1.6-400 | 100 | 40 | 3.2444 | 5.2500 | 102 |
| ZnAl-1.7-400 | 100 | 37 | 3.2471 | 5.2075 | 51 |
| ZnAl-1.8-400 | 100 | 39 | 3.2408 | 5.2178 | 40 |
| ZnAl-2.6-400 | 100 | 37 | 3.2539 | 5.2181 | 64 |
| ZnAl-3.4-400 | 100 | 45 | 3.2605 | 5.2095 | 77 |
| ZnAl-4.4-400 | 100 | 47 | 3.2554 | 5.2137 | - |
| ZnAl-1.9U-400 | 100 | 21 | 3.2191 | 5.1248 | 110 |
| ZnAl-1.9K-400 | 100 | 118 | 3.2526 | 5.2071 | 106 |

**
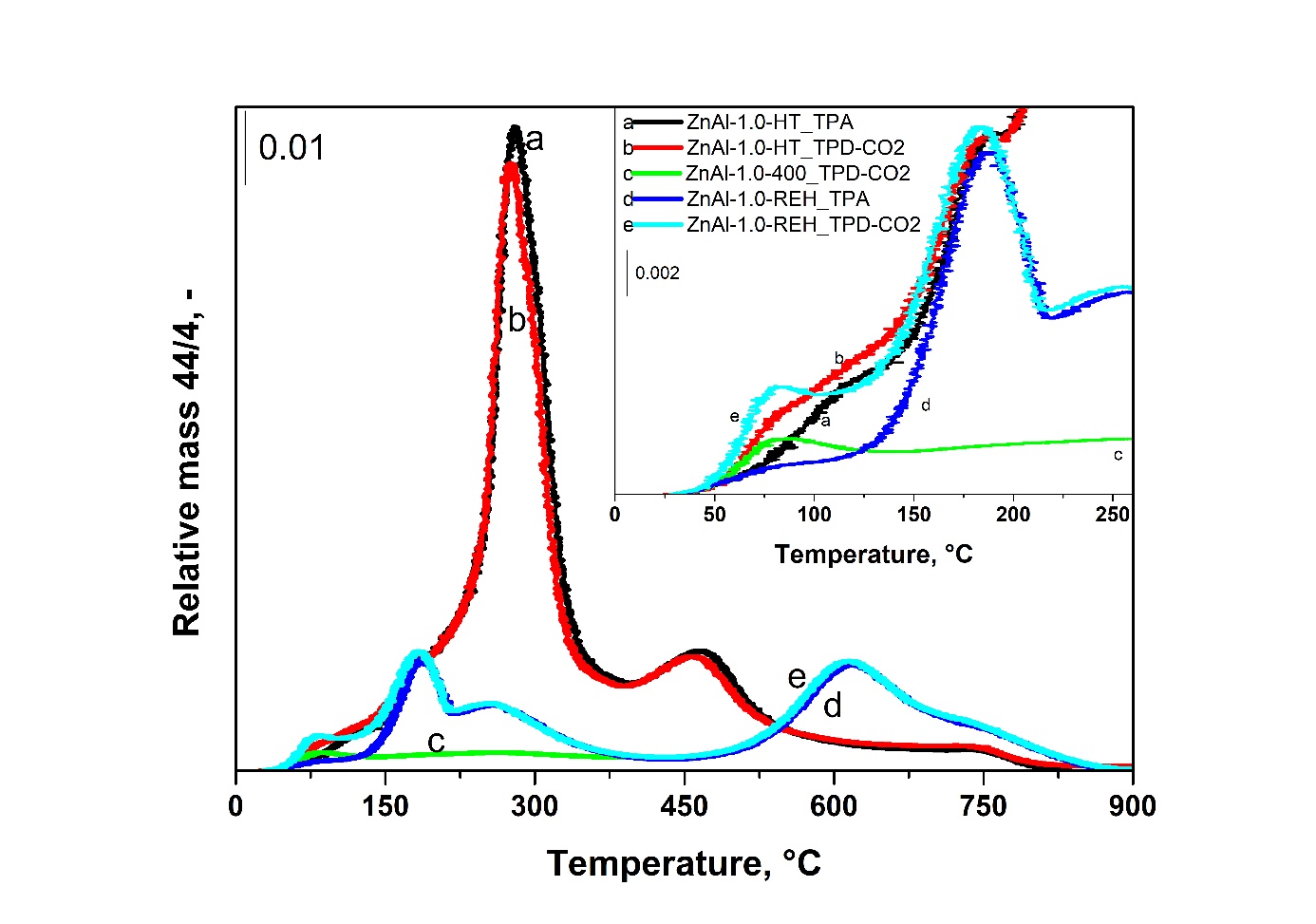
Fig S2A** TPD-CO_2_ profiles of ZnAl-1.0-HT hydrotalcite, ZnAl-1.0-400 mixed oxide, ZnAl-1.0-REH reconstructed LDH and TPA (experiment without CO_2_ adsorption) profiles of ZnAl-1.0-HT LDH and ZnAl-1.0-REH RE-LDH.

**
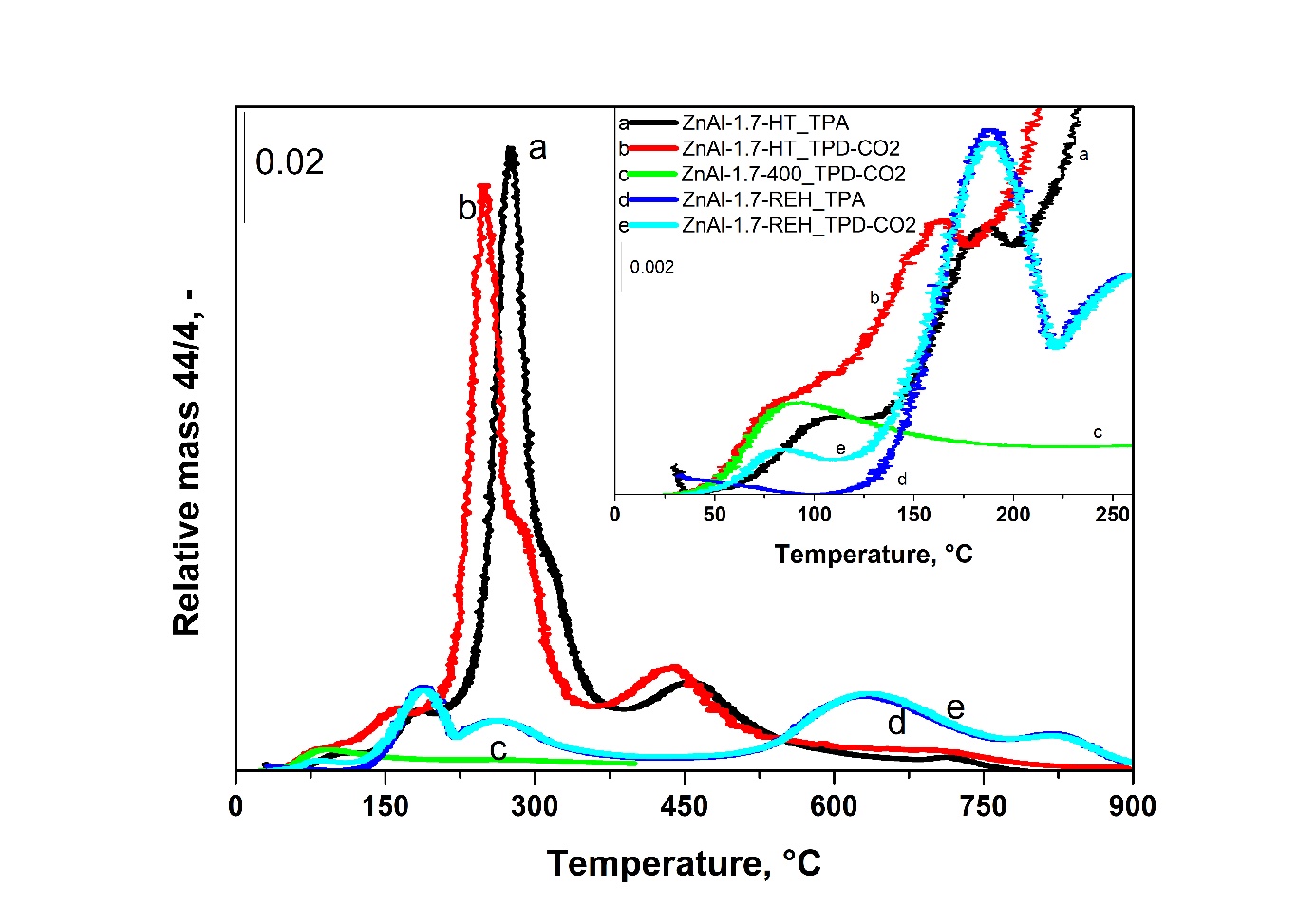
Fig S2B** TPD-CO_2_ profiles of ZnAl-1.7-HT hydrotalcite, ZnAl-1.7-400 mixed oxide, ZnAl-1.7-REH reconstructed LDH and TPA (experiment without CO_2_ adsorption) profiles of ZnAl-1.7-HT LDH and ZnAl-1.7-REH RE-LDH.

**
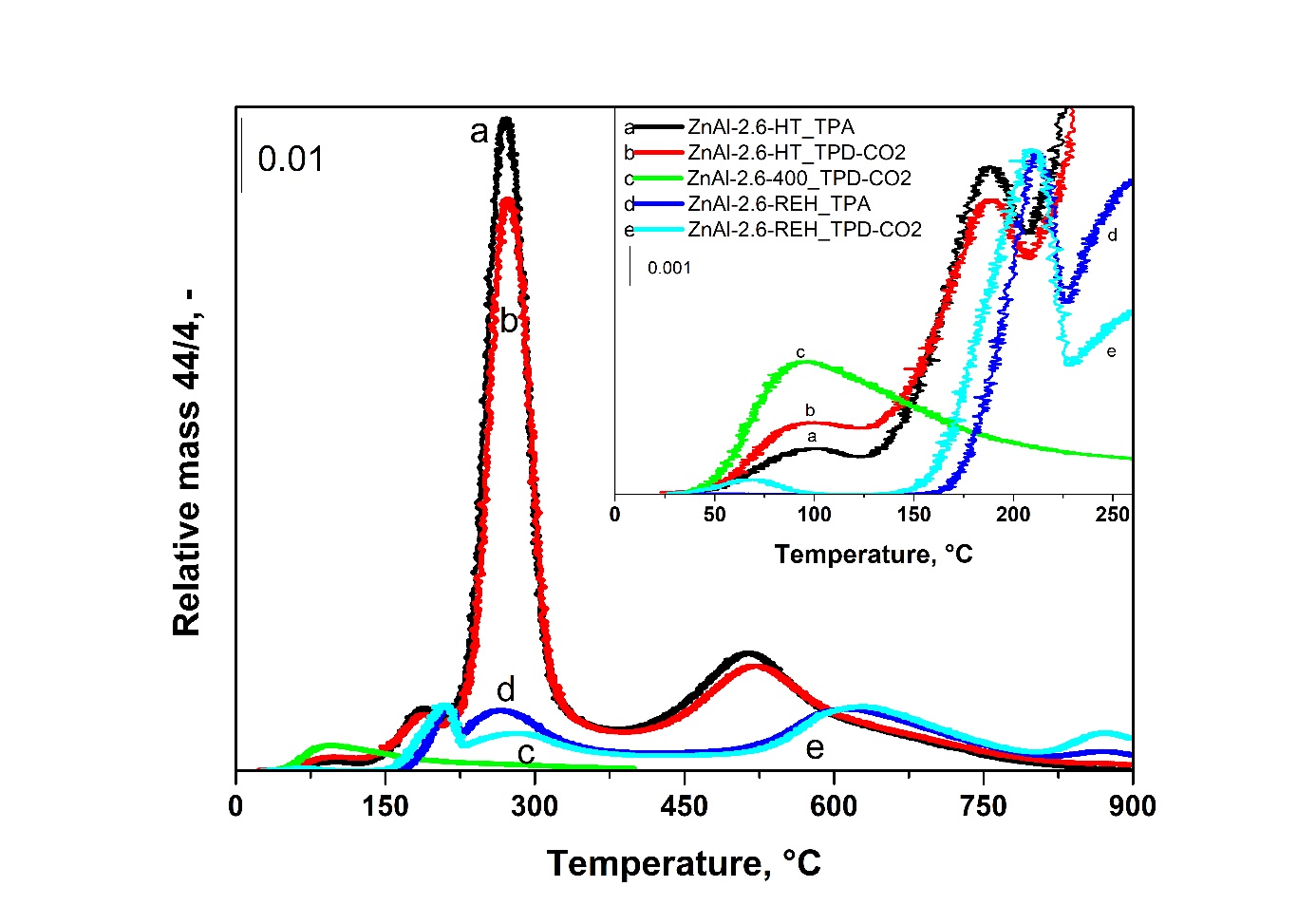
Fig S2C** TPD-CO_2_ profiles of ZnAl-2.6-HT hydrotalcite, ZnAl-2.6-400 mixed oxide, ZnAl-2.6-REH reconstructed LDH and TPA (experiment without CO_2_ adsorption) profiles of ZnAl-2.6-HT LDH and ZnAl-2.6-REH RE-LDH.

**
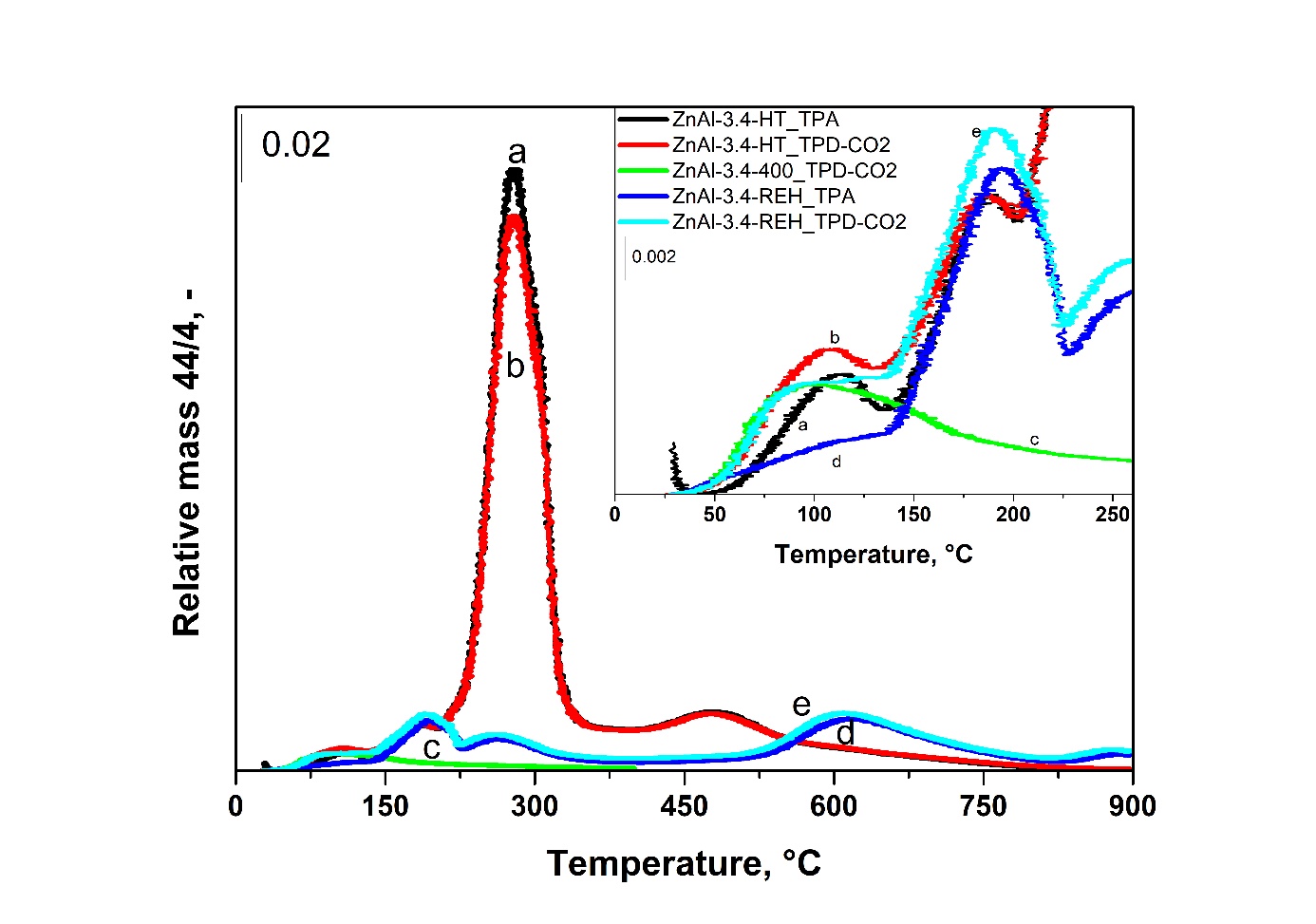
Fig S2D** TPD-CO_2_ profiles of ZnAl-3.4-HT hydrotalcite, ZnAl-3.4-400 mixed oxide, ZnAl-3.4-REH reconstructed LDH and TPA (experiment without CO_2_ adsorption) profiles of ZnAl-3.4-HT LDH and ZnAl-3.4-REH RE-LDH.

**
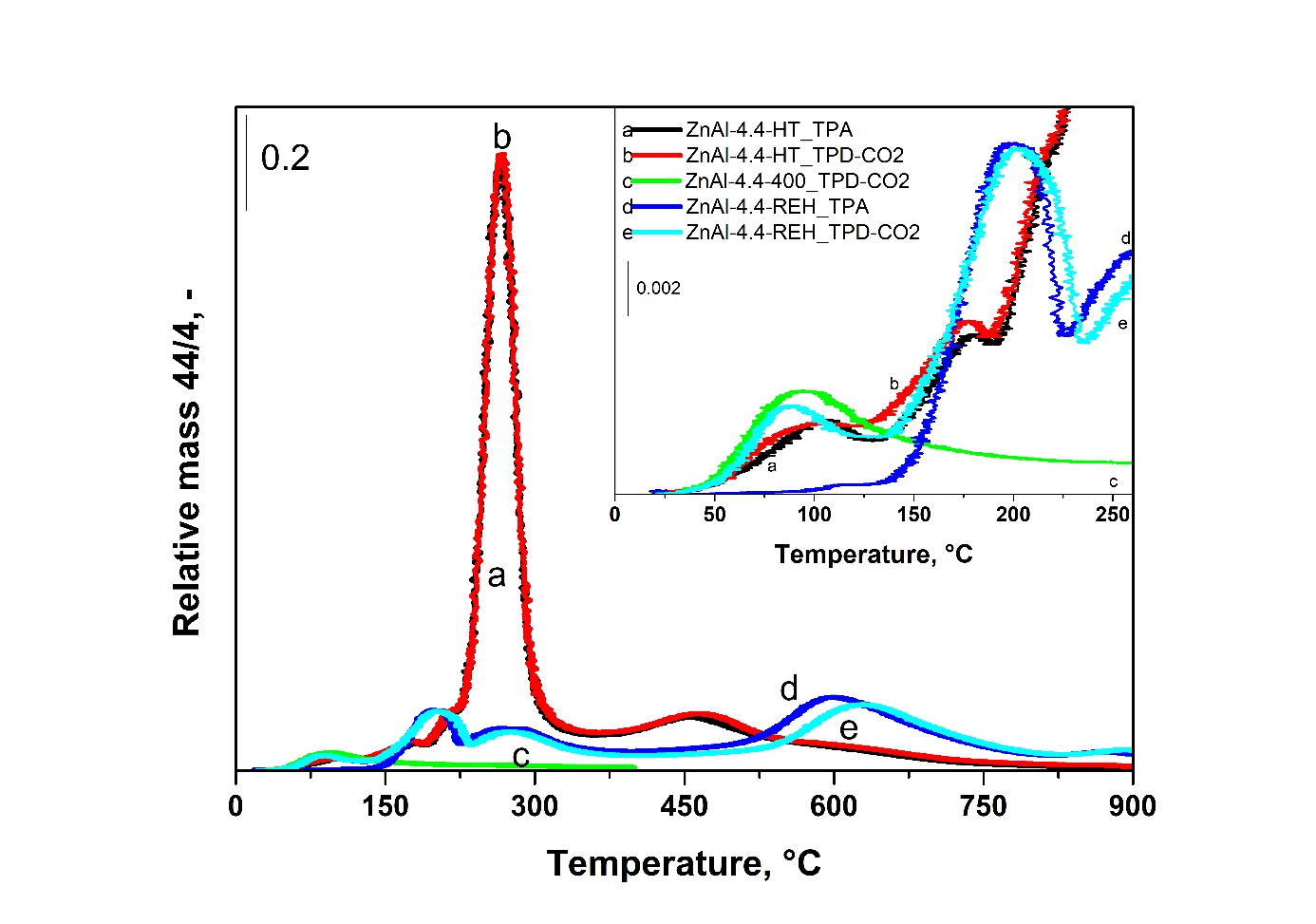
Fig S2E** TPD-CO_2_ profiles of ZnAl-4.4-HT hydrotalcite, ZnAl-4.4-400 mixed oxide, ZnAl-4.4-REH reconstructed LDH and TPA (experiment without CO_2_ adsorption) profiles of ZnAl-4.4-HT LDH and ZnAl-4.4-REH RE-LDH.

**
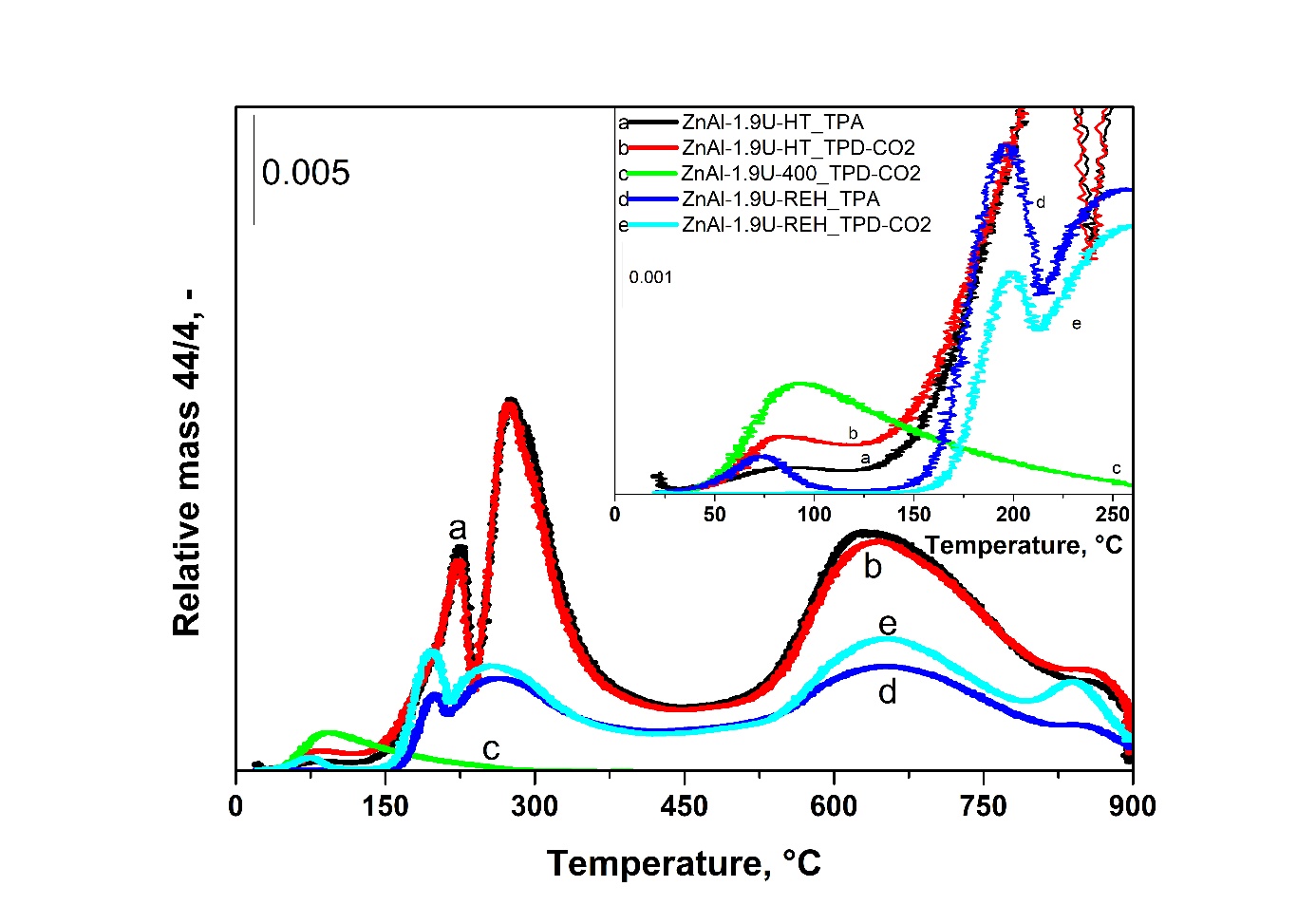
Fig S2F** TPD-CO_2_ profiles of ZnAl-1.9U-HT hydrotalcite, ZnAl-1.9U-400 mixed oxide, ZnAl-1.9U-REH reconstructed LDH and TPA (experiment without CO_2_ adsorption) profiles of ZnAl-1.9U-HT LDH and ZnAl-1.9U-REH RE-LDH.

**
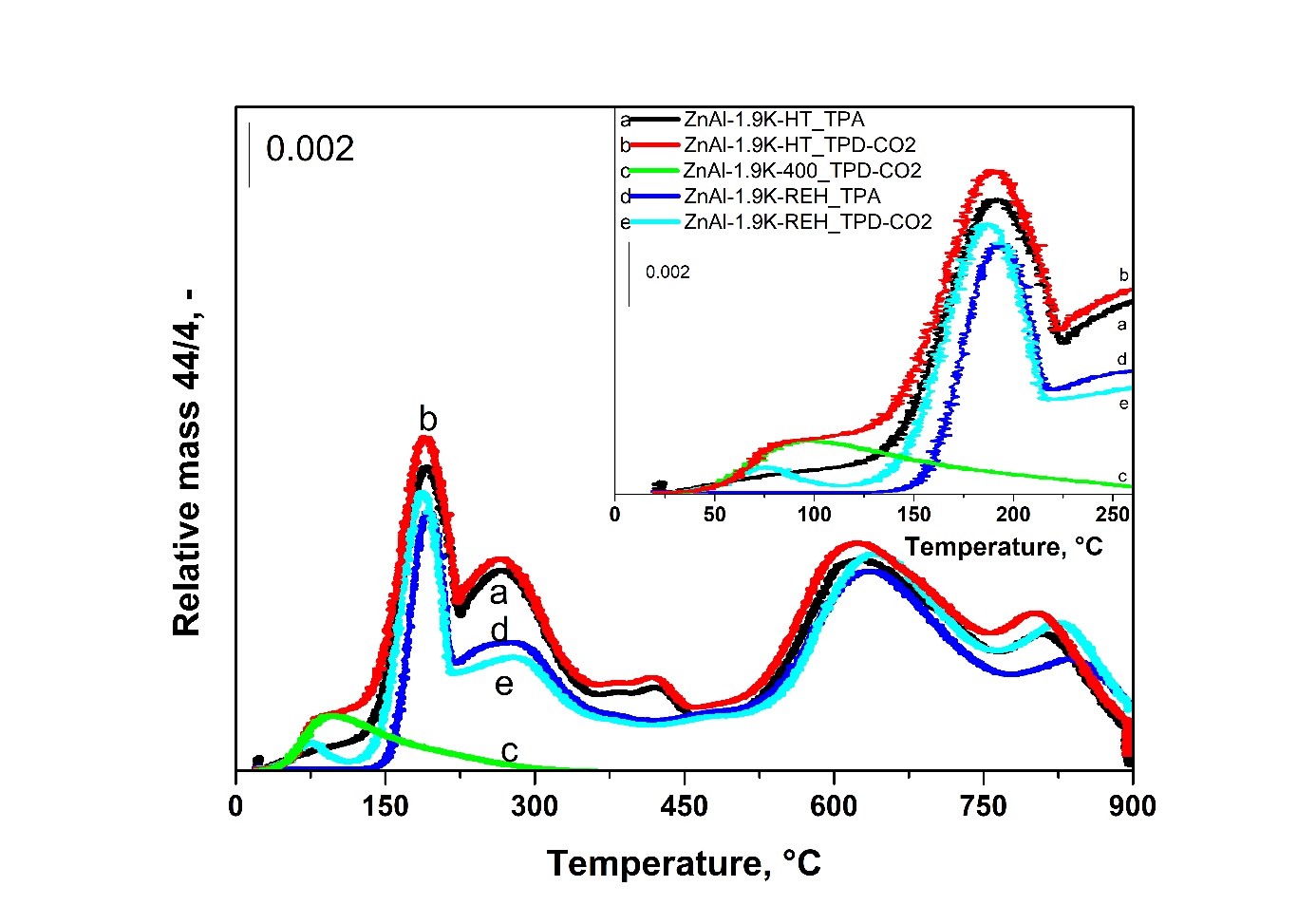
Fig S2G** TPD-CO_2_ profiles of ZnAl-1.9K-HT hydrotalcite, ZnAl-1.9K-400 mixed oxide, ZnAl-1.9K-REH reconstructed LDH and TPA (experiment without CO_2_ adsorption) profiles of ZnAl-1.9K-HT LDH and ZnAl-1.9K-REH RE-LDH.
